# The Effect of *Lactiplantibacillus plantarum* and *Lacticaseiba-cillus Rhamnosus* Strains on the Reduction of Hexachlorobenzene Residues in Fermented Goat Milk During Refrigerated Storage

**DOI:** 10.3390/molecules29235686

**Published:** 2024-11-30

**Authors:** Agata Witczak, Izabela Dmytrów, Anna Mituniewicz-Małek

**Affiliations:** Department of Toxicology, Dairy Technology and Food Storage, Faculty of Food Sciences and Fisheries, West Pomeranian University of Technology, 70-310 Szczecin, Poland; izabela.dmytrow@zut.edu.pl (I.D.); anna.mituniewicz-malek@zut.edu.pl (A.M.-M.)

**Keywords:** goat milk, hexachlorobenzene, probiotic bacteria strains, refrigerated storage

## Abstract

Hexachlorobenzene (HCB) is a persistent organic pollutant (POP) commonly detected in milk and dairy products. These compounds pose a serious threat to the health of consumers due to their considerable bioaccumulation potential, high stability, and toxicity. (2) Methods: The study evaluated the potential of *Lactiplantibacillus plantarum* and *Lacticaseibacillus rhamnosus* probiotic cultures to reduce HCB residues in fermented goat milk beverages during 21-day refrigerated storage. HCB content was determined by gas chromatography coupled with mass spectrometry (GC/MS). (3) Results: A strong negative correlation was found between HCB concentration in fermented milk and storage time. After 21 days, a 75–78% reduction in HCB content was observed, with *L. plantarum* showing greater efficiency in reducing hexachlorobenzene levels than *L. rhamnosus.* (4) Conclusions: The use of probiotic cultures contributed to a significant reduction in the HCB content of fermented goat milk. Our findings support the hypothesis that the lactic acid bacteria *Lactiplantibacillus plantarum* and *Lacticaseibacillus rhamnosus* can lower hexachlorobenzene levels in fermented products

## 1. Introduction

Currently, around 18.6 million tonnes of goat milk are produced a year. Of this, more than 50% come from Asia, with the largest producers being India, Pakistan, and Bangladesh [1]. In Europe, goat milk production is around 2.8 million tonnes, of which more than 78% (2.2 million tonnes) is farmed within the European Union, with Greece having the largest share [2]. In 2019, sales were projected to generate revenues of about $15 billion by 2024, with a 53% rise by 2030 [3]. There is clearly growing interest in both goat milk and its products [4].

Goat milk has a similar chemical composition to cow milk, typically containing 12.7% dry matter, 87.3% water, 0.8% ash, 2.9% protein, and 3.6% fat [1,5]. However, goat milk is characterized by a higher degree of fat dispersion, smaller fat globules, and a higher content of short-chain fatty acids, which positively affects digestion and assimilation [6]. Goat milk also possesses higher concentrations of polyunsaturated fatty acids (PUFAs), particularly linoleic acid (LA, +52.8%) and docosahexaenoic acid (DHA, +123.8%), and lower concentrations of saturated fatty acids (SFAs) such as myristic acid (C14:0, −6.8%), palmitic acid (C16:0, −8.5%), and stearic acid (C18:0, −8.7%). It also possesses lower concentrations of trans-isomeric fatty acids, including monounsaturated (MUFA, −20.9%), polyunsaturated (PUFA, −82.6%), and total fatty acids (FA, −21.6%) [7].

In addition, goat milk contains at least 16% less casein and at least 9% less lactose compared to cow milk. Between 0 and 25% of the total casein content in goat milk is αS1-casein, compared to as much as 40% in cow milk [8]. In addition, the αS1-casein content in goat milk is further reduced by pasteurization. Otherwise, the two milk types have similar levels of other casein fractions, with a slightly higher β-casein content in goat milk (+11–18%).

Goat milk is also rich in calcium (Ca), phosphorus (P), magnesium (Mg), potassium (K), and sodium (Na) [9], although the content of these minerals differs between the colloidal and soluble phases: the colloidal phase is the main source of calcium, copper (Cu), iron (Fe), zinc (Zn), phosphorus, and manganese (Mn), while the soluble phase provides magnesium, sodium, and potassium [10]. It also provides higher calcium (134 mg/L), potassium (181 mg/L), phosphorus (121 mg/L), iron (0.07–1.025 mg/L), magnesium (16 mg/L), copper (≥0.05 mg), zinc (0.56 mg), manganese (0.032 mg), and selenium bioavailability (1.33 mg/L) compared to cow milk [1,11]. However, it also contains less sulphur (28 mg/L) and sodium (41 mg/L) [1].

The vitamins in goat milk demonstrated higher bioavailability than in cow milk. Goat milk contains more thiamine, riboflavin, niacin, pyridoxine, and vitamins A, C, and D but lower levels of biotin, folic acid (up to five times) and cobalamin. Depending on the breed of goat, the vitamin A content ranges from 0.255 to 0.398 mg/L, vitamin E from 0.764 to 1.209 mg/L, and B2 from 0.702 to 1.239 mg/L [5,12].

The consumption of goat milk has been associated with various health benefits, partly due to the presence of compounds with antioxidant properties and bioactive peptides. Its antioxidant activity is approximately 74%, as determined by ABTS, and the total polyphenol content ranges from 54.7 to 59.3 mg TAE/100 mL [6]; these compounds can neutralize free radicals, which helps to prevent cancer and delay ageing processes [13]. Mejia Palma et al. [14] report that certain peptides present in goat milk act as angiotensin-converting enzyme (ACE) inhibitors, which can lower blood pressure. In addition, drinking goat milk has been found to increase the number of bacteria from the genera *Lactobacillus* and *Muribaculum* [15] in the human intestinal microbiota.

Goat milk also exhibits immunomodulatory properties due to, inter alia, the presence of proteins such as lactoferrin and casein and its ability to reduce the levels of pro-inflammatory cytokines. The components of goat milk also have anti-atherosclerotic and anti-diabetic effects [16,17].

Although fermented goat milk has poor rheological properties compared to cow milk, such as less firmness, poorer texture, and greater susceptibility to syneresis [18], its health benefits have led to it being increasingly used in the production of fermented foods, i.e., yoghurt, acidophilic milk, kefir, kumis, or cheese. In addition, goat milk has been found to be an excellent carrier of probiotic bacteria in the human diet [3].

Unfortunately, due to their lipophilic nature and their ability to bioaccumulate in living tissues, persistent organic pollutants (POPs), such as the pesticide hexachlorobenzene (HCB), are still found in a variety of foods, including fish, poultry, milk, and dairy products.

Previous studies indicate that HCB is widely distributed throughout the marine environment, mainly in fish and seafood (Table 1). It is believed to enter rivers primarily as a pesticide, and from there, it passes into seas and oceans. HCB is now ubiquitous and is believed to move between ecosystems. Moon et al. [19] reports its presence at levels ranging from 5.8 to 573 pg/g in fish and seafood in the coastal waters of Korea despite it not being used in the region; however, the authors indicate these concentrations are not hazardous to human health [19].

The presence of HCB in human milk is a significant problem as younger consumers are particularly vulnerable [20,21]. In Ghana, where pesticide use is mostly used in agriculture, environmental HCB levels have declined significantly over the past three decades due to bans on HCB emissions and production. Indeed, although HCB is universally present in the human body due to both diet and environmental pollution, the amounts can vary considerably within countries or regions [19].

**Table 1:** Examples of levels of HCB in various grocery products.

Country	Material	Amount of HCB	Author
Egypt	Buffalo milk	<0.200	Shaker and Elsharkawy (2015) [22]
	Mussels	<LOD 0.50 µg/g dw	Khairy et al. (2012) [23]
	Potato tubers (skin and pulp)	0.014, <0.026	Soliman (2001) [24]
	French fries	0.006, <0.011	Soliman (2001) [24]
	Potato chips	0.003, <0.006	Soliman (2001) [24]
Ghana	Fish (Tilapia and Suma)	2.10	Adu-Kumi et al. (2010) [25]
Tanzania	Tilapia (*Oreochromis* sp.)	0.6–4.0	Polder et al. (2014) [26]
Tunisia	Common sole (*Solea solea*)	1.27–15.1	Ben Ameur et al. (2013) [27]
	Common Cephalus (*Mugil cephalus*)	1.62–28.5	Ben Ameur et al. (2013) [27]

In a study of livestock adipose tissue, Hamadamin and Hassan [28] report the mean HCB content to be 0.236 ng/g in cattle, 0.185 ng/g in sheep, and 0.114 ng/g in goats. In contrast, Dokić et al. [29] found HCB concentrations of 0.92 ng/g w.w. (wet weight) in raw cow milk, 1.03 ng/g w.w. in sheep milk, and 1.70 ng/g w.w. in raw goat milk. In contrast, Derouiche et al. [30] identified HCB in nearly 77% of tested cow milk samples. Moreover, HCB was also detected in breast milk (mean 570 ng/g lipid), which can pose a serious health risk to breastfed infants [31,32].

Hexachlorobenzene (HCB) is a persistent, non-degradable, chlorinated hydrocarbon that was first introduced as a fungicide in 1945 to fix seeds for sowing [33]. Despite being banned in most countries in the late 1970s and early 1980s, the compound remains ubiquitous in the environment and poses a real threat to health. On the basis of Commission Regulation EC 1272/2008 [34], HCB is classified as a carcinogen in hazard category 1B, with the notation H350, meaning that it may cause cancer.

According to the toxicokinetic properties of hexachlorobenzene, it is partially absorbed in the gastrointestinal tract and readily distributed throughout the body, preferentially reaching fatty tissues. It also easily crosses the placenta. It is metabolized by microsomal enzymes in the liver, kidneys, lungs, and intestines. Hexachlorobenzene is slowly metabolized in the liver to form pentachlorophenol, pentachlorobenzene, tetrachlorobenzene, and several unidentified compounds. In humans, hexachlorobenzene is excreted mainly in urine in the form of metabolites, pentachlorophenol, and pentachlorothiophenol. In animals, hexachlorobenzene absorbed orally is primarily excreted unchanged through the feces. Hexachlorobenzene also concentrates in milk, including human milk.

The Commission Regulation (EU) 2016/1866 [35] sets maximum residue levels (MRLs) for this compound in various foods, with 0.005 mg/kg for goat milk. HCB exposure has been associated with i genetic mutations, tumours, birth defects, neurological disorders, liver disease, and skin lesions and hyperpigmentation [36]. The presence of HCB in the body also leads to endocrine system dysfunction. It can alter the activity of 5′-deiodinase in the liver and thyroid gland, an enzyme that plays a key role in thyroid hormone metabolism. As reported by Dou et al. [37], HCB exposure caused oxidative damage in the testes of mice and led to a decrease in spermatogenesis, with the toxicity of the compound not being dose dependent. HCB administration to rats significantly increased blood urea nitrogen, serum creatinine, cholesterol, and phospholipids, indicating HCB-induced nephrotoxicity and hepatotoxicity [38]. HCB induced apoptosis in rat hepatocytes and alveolar cells. In addition, the compound caused significant intestinal and brain damage due to oxidative stress. The threat posed by the presence of hexachlorobenzene in the environment necessitates the continuous search for methods of its neutralization or degradation. Previous studies have primarily focused on the potential for biodegradation of HCB by soil microorganisms to remediate soils contaminated with persistent organic pollutants (POPs) [39]. The growth in consumer awareness regarding the health benefits of probiotics for directly enhancing the health value of food products, by enriching gut microbiota and increasing their ability to metabolize various compounds, has driven research into the use of probiotic strains for the biodegradation of HCB residues in food.

Accordingly, in the present study, it is hypothesized that the probiotic strains *Lacticaseibacillus rhamnosus* LCR (Lactoferm LCR Pro-TekR) and *Lactiplantibacillus plantarum* subsp. *plantarum* LP (Lactoferm LP Pro-TekR) (Biochem S.r.l., Monterotondo, Rome, Italy) can effect changes in the HCB content of fermented goat milk. It was also hypothesized that the duration of refrigerated storage may influence the extent of these changes. The aim of the study was to determine the influence of the probiotic cultures *L. rhamnosus* and *L. plantarum* on changes in HCB content in fermented goat milk stored under refrigerated conditions (5 °C ± 1 °C) for 21 days. This work represents a continuation of previous research presented in [39].

## 2. Results

### 2.1. pH and Dry Matter Content in FGM 

The dry matter content in FGM ranged from 9.73% to 10.28%; no significant changes were observed regarding storage time or fortification with HCB. The highest pH value was recorded on the first day of refrigeration in the Mix_HCB_ variant (4.53 ± 0.02), while the lowest was noted in the Mix variant (4.48 ± 0.01), with greater pH fluctuations observed in the HCB-fortified samples. After 21 days of refrigeration, the greatest decrease in pH was recorded for the Mix_HCB_ variant (4.26 ± 0.02) and the least for the Mix variant (4.28 ± 0.01). In most cases, the type of culture or the addition of HCB had significant influence on changes in pH (*p* < 0.05); however, it was noted that the pH of the FGM fell proportionally with percentage of the HCB content, and it was inversely proportional to the refrigeration storage time (Table 2).

The observed decrease in the pH of the samples over the storage period is consistent with expectations. This phenomenon results from the conversion of lactose to lactic acid. The production of lactic acid during lactic fermentation begins with the addition of a starter culture to the milk. Changes in the acidity of the milk during fermentation result from the enzymatic activity of lactic acid bacteria introduced in the form of a starter. These bacteria hydrolyze lactose into d-glucose and d-galactose under the influence of β-d-galactosidase. Further conversion of glucose to lactic acid during fermentation leads to the acidification of the environment and a decrease in pH. Depending on the type of product, acidity serves as a key indicator, reflecting both the freshness of the product and the correctness of the acidification process occurring during storage. Lactose conversion also takes place during product storage.

The quality of fermented milk, and thus its consumer acceptability, is largely determined by the degree of coagulum acidification. Changes in acidity result from the presence of starter bacteria, which exhibit varying levels of acidifying, proteolytic, and lipolytic activity. During the fermentation and storage process, these bacteria utilize milk components, albeit at different rates and to different extents. The metabolic processes initiated during the incubation of inoculated milk primarily lead to a gradual reduction in lactose content, which may decrease by several to even tens of percentage points. As the lactose content in fermented milk decreases, there is a concurrent increase in lactic acid content [40]. Improper storage conditions, leading to excessive acidification of the product, consequently result in the deterioration of its structure, a reduction in viscosity, and an increased susceptibility to syneresis [41,42]. Some starter microorganisms can survive during refrigerated storage, transportation, and sales, and their metabolism leads to the excessive accumulation of lactic acid. This can result in over-acidification of the product, causing high acidity, dehydration of the curd, intense whey separation, off-flavors, and a reduction in the number of viable LAB cells [43]. The acidity of the fermented milk studied was typical for this type of product.

### 2.2. Changes in HCB Content During Refrigerated Storage of Fermented Goat Milk 

**Figure 1:**
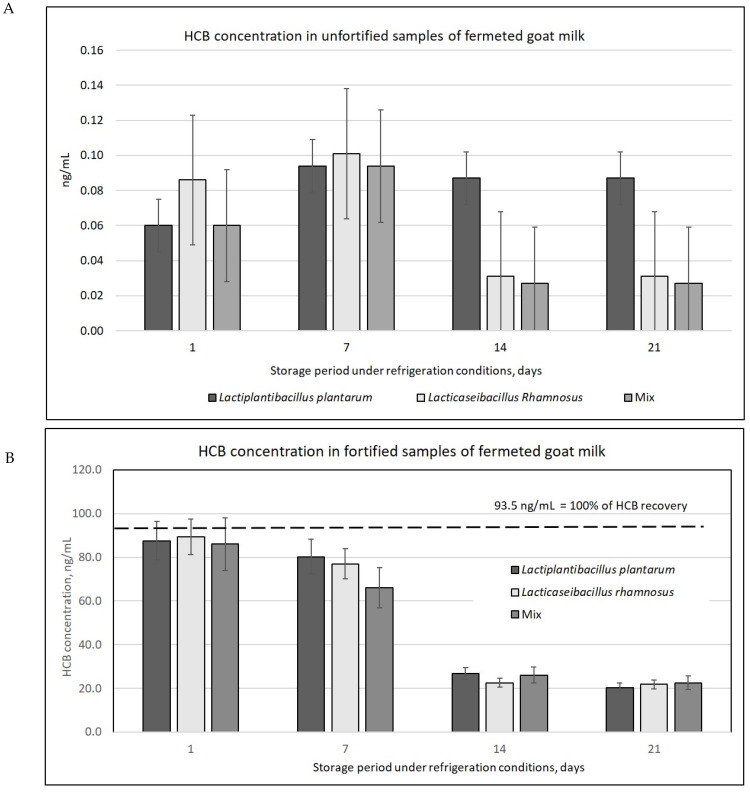
Concentration of HCB in unfortified (**A**) and in fortified (**B**) samples of fermented goat milk (FGM), depending on the culture. The bars: arithmetic means with standard deviations.

Goat milk, used as a raw material to produce fermented milk, exhibited a low HCB content, averaging 0.09 ± 0.01 ng/mL. Similarly, HCB levels were found to be equally low in unfortified FGM (Figure 1A). The concentration of this pesticide varied during storage; however, to observe a correlation, it was necessary to prepare samples fortified with the analyzed compound.

To eliminate errors and accurately determine the extent of HCB loss during storage, residual HCB levels in unfortified samples were removed from those in spiked samples. In this way, an enrichment recovery value of 93.5 ppb was obtained, in relation to which the percentage loss of HCB was determined in subsequent periods of refrigerated storage.

The HCB content of the FGM changed during refrigeration, with a decrease being apparent after seven days of storage. In the treated FGM, significant (*p* < 0.05) changes in HCB concentration were observed during storage compared to the baseline pesticide content, i.e., 93.5 ng/mL (100%).

**Figure 2:**
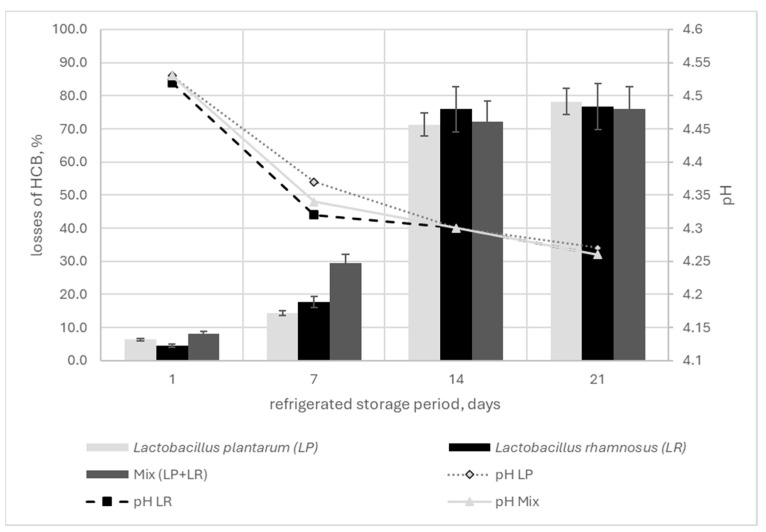
Effect of storage period on changes in HCB (%) and pH of fermented goat milk (FGM), depending on the culture. The bars: arithmetic means with standard deviations.

**Figure 3:**
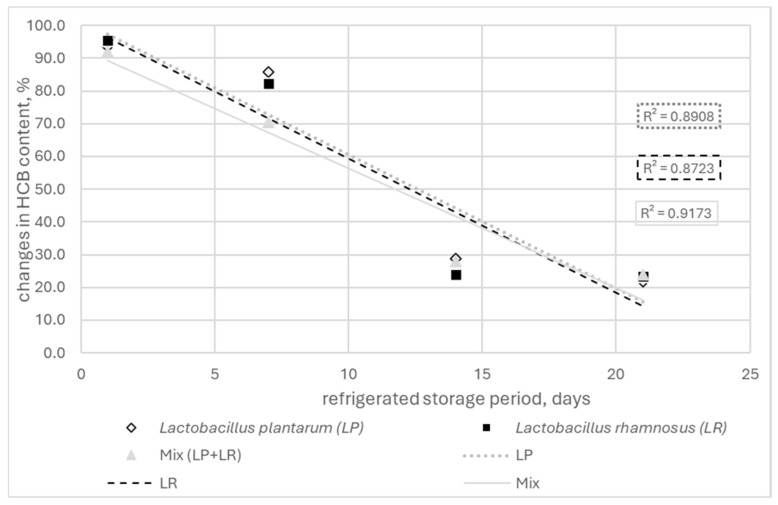
Correlation between refrigeration storage time and changes in %HCB content in fermented goat milk (FGM).

A significant relationship was found between refrigerated storage time and change in HCB content (*p* < 0.05; Duncan’s test). The correlation between these parameters was positive and very strong (r = 0.898–0.790) (Figure 1, Figure 2 and Figure 3). Also, the type of probiotic bacteria had little effect on the changes in HCB content, with a 71.3 (LP) 75.9% (LR) reduction observed after 14 days and a 75.9 (Mix) 78.2% (LP) after 21 days (Table S1).

## 3. Discussion

The widespread use of HCB in the second half of the 20th century led to its accumulation in the environment and, consequently, in raw materials and food products [28,29,30,39]. Due to the increasing awareness of ongoing exposure to HCB, scientists have been and continue to explore methods to reduce its levels. One promising approach is microbial biodegradation. The research conducted in this study, along with findings from various other authors, suggests the potential use of microorganisms to reduce or alter the structure of toxic compounds in different matrices, ranging from water, soil, and air to animal and plant tissues.

An example of such research is the work of Wang et al. [44], who investigated the potential of using rhizoremediation to lower HCB levels in water. Rhizoremediation is a biotechnological method where plants and the microorganisms living in their roots (the rhizosphere) assist in the degradation, detoxification, or removal of pollutants from soil, water, or air. The authors confirmed that the perennial herb *T. angustifolia* aids in the degradation of HCB in water, with the initial concentration of HCB being the primary factor responsible for this process. The most effective bacteria in adapting to HCB contamination were from the genus *Bacillus*, while the dominant bacterial types in HCB-contaminated water were *Proteobacteria* and *Firmicutes*. Genetic studies of bacterial structure revealed that both the rhizosphere and HCB concentrations significantly influence bacterial communities, suppressing some populations while promoting others [44].

Similarly, Da-Zhong et al. [45] reported that under methanogenic conditions, modified *Escherichia coli* DH5α and the natural PCP degrader *Sphingobium chlorophenolicum* ATCC 39,723 degrade PCP into pentachlorobenzene (QCB), which then breaks down into tetra-, tri-, and dichlorobenzenes and chlorobenzene. Additionally, Yan et al. [46] demonstrated that *S. chlorophenolicum* can completely reduce HCB, with nearly 40% of the compound being degraded within 24 h. Dimova et al. [47] achieved a 69% reduction in HCB using bacteria from the *Comamonas testosteroni* genus.

Research on pesticide biodegradation increasingly focuses on lactic acid bacteria (LAB). These bacteria have been found to reduce the concentration of organophosphorus pesticides in milk by up to 64.6%, with the highest efficacy observed in *Lactobacillus bulgaricus*, *Lactobacillus rhamnosus*, and *Lactobacillus thermophilus* strains [46]. Witczak et al. [39] confirmed that LAB can lower HCB levels in mare milk by 73.8% to 78.8%. Our current results also indicate the potential for HCB reduction in FGM. Although complete elimination of HCB was not fully achieved, an average reduction of 75% was observed.

The raw goat milk used in the studies described here had a low HCB content. The maximum HCB concentration in both raw and FGM was 0.09 ± 0.01 ng/mL, which is approximately 2% of the maximum residue level (MRL) of 0.005 μg/g. The MRL value is about 50 times higher than the results obtained for dairy products from goat milk. To assess the impact of probiotic bacteria on HCB degradation, HCB was added to the raw milk as described in the “Materials and Methods” section. The high HCB content in the fermented goat milk resulted from this addition, not from the initial HCB content in the milk.

Petrova et al. (2022) reported that LAB from the genera *Lactobacillus* and *Leuconostoc* can metabolize a wide range of pesticides, utilizing them as sources of carbon and energy. The degradation mechanism relies on esterase and phosphatase enzymes [47]. *L. plantarum* successfully degraded pesticides, such as malathion, methyl chlorpyrifos, and deltamethrin in sauerkraut and fermented olives, achieving a pesticide reduction of 50–60% [48,49]. The most described mechanism of pesticide elimination by LAB is enzymatic hydrolysis, involving carboxylesterases, organophosphorous hydrolases, phosphotriesterases, and phosphatases.

LAB are increasingly recognized as effective detoxifying agents for food contaminated with pesticides, including organochlorine, organophosphorus, and pyrethroid pesticides. Fermentation is also regarded as an effective method for reducing pesticide levels [50]. Probiotics possess genes that encode enzymes responsible for pesticide degradation [51]. Lili et al. [52] found that organophosphorus pesticides are degraded by phosphatase enzymes produced by LAB, which convert these pesticides into dialkyl phosphate and aryl alcohol in the presence of water. Duan et al. [53] demonstrated that fermentation reduced the residual concentration of organochlorine pesticides in yogurt, with a processing factor ranging from 0.42 to 0.64. Organochlorine pesticides are lipophilic, and an increase in HCB concentration can be observed as the fat content in the product rises, particularly during whey production and maturation [53]. However, no significant changes in water content were observed during the three-week storage of fermented goat milk that could have affected HCB concentration. The bacterial starter culture must ensure a minimum therapeutic level, with probiotic survival rates exceeding 10^6^ CFU/g. Probiotic bacteria remain viable under refrigerated conditions, suggesting that enzymatic activity at low temperatures could be responsible for HCB degradation. After lactose digestion, HCB may become an alternative carbon source.

Our findings confirm those of other researchers indicating that microorganisms can be utilized to reduce the levels of toxic compounds or alter their structure in a variety of materials, including water, soil, air, and animal or plant tissues. Since nutritionally valuable nutrients ingested through food are often accompanied by unnecessary or even toxic compounds, there is a pressing need to design reliable methods for their elimination. This is particularly relevant for individuals living in areas where the primary income is derived from heavy industry, which remains a source of substances such as HCB.

The use of probiotic bacterial monocultures or mixtures of probiotic bacteria resulted in significant (*p* < 0.05) reductions in HCB content in fermented goat milk (FGM) during storage. All inoculation cultures achieved similar percentage reductions in HCB after 21 days of storage, ranging from 75% to 78%, with the *Lactiplantibacillus plantarum* monoculture showing marginally greater effectiveness. Additionally, strong positive correlations were observed between acidity and changes in HCB content among the FGM variants during refrigerated storage (r = 0.79–0.89).

The results obtained in this study also suggest the need for further research into the analysis of metabolites produced in food.

## 4. Materials and Methods

### 4.1. Test Material

Fermented goat milk (FGM) was prepared by the thermostat method under laboratory conditions. Goat milk (density: 1.0245 g/cm^3^, lactose content: 4.5%, protein: 4.0%, fat: 3.5%, dry matter: 10.4%, pH: 6.72, titratable acidity: 6 °SH) was purchased from an organic farm Kozi Gródek near Szczecin. After transport to the laboratory, the milk was pasteurized using the tank method (85 ± 1 °C, 30 min) and then cooled to the inoculation temperature with starter cultures (42 ± 1 °C). The cooled milk was divided into two batches: one with HCB and one without. The first batch was supplemented with a standard solution (certified reference material, Supelco 40008, Darmstadt, Germany) containing a known amount of HCB (93.5 ng/mL); this batch would be used to track changes in HCB content during storage. After the addition of HCB, the material was homogenized. The second batch was not amended with HCB and was used to prepare control samples.

**Figure 4:**
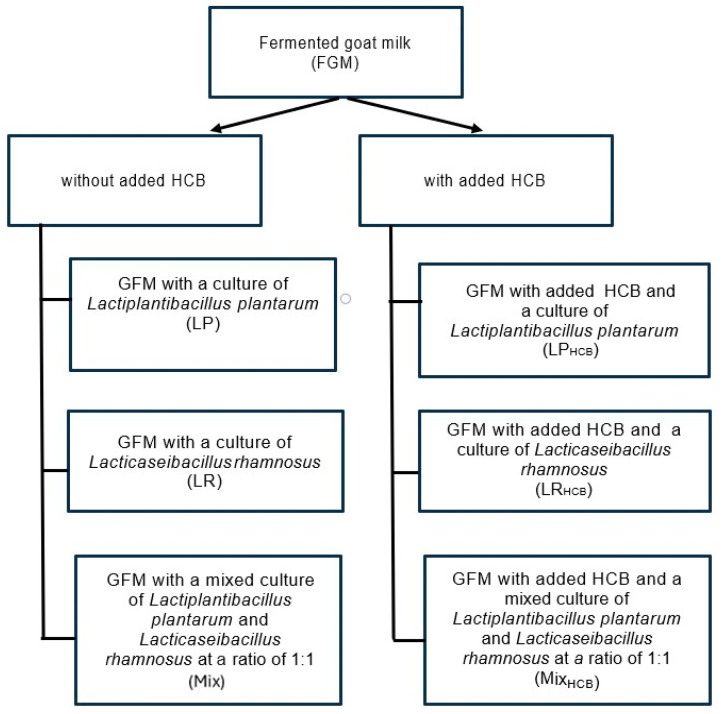
The variants of fermented milk.

All FGM variants were prepared by adding a 7% (*v*/*v*) starter made with probiotic starters to both batches of the processed milk. The inoculated milk was poured into cups (100 mL each), hermetically sealed, coded, and placed in an incubation chamber for fermentation. The end of fermentation was determined by measuring the pH of the samples and comparing the value with the fermentation curve included in the culture specification [39]. The starter culture was obtained by reviving previous cultures according to the manufacturer’s specifications: briefly, a portion of the culture (0.6 g/L) was incubated in skimmed milk (0.0%) for four to eight hours at 40 °C. The following concentrated freeze-dried probiotic cultures were used in the experiment: *Lactiplantibacillus plantarum* LP (Lactoferm LP Pro Tek^®^; Biochem s.r.l., Modena, Italy) and *Lactocaseibacillus rhamnosus* LR (Lactoferm LCR Pro Tek^®^; Biochem s.r.l., Modena, Italy). Six FGM variants were prepared (Figure 4): three variants were fortified with a known amount of HCB (93.5 ppm) and three reference variants were not fortified.

In order to trace the changes in HCB content, random samples were taken at seven-day intervals, i.e., on day 1, 7, 14, and 21 of storage (5 °C ± 1 °C). Each time, the dry matter content and pH were determined, after which the samples were frozen (−21–1 °C) for further analyses.

### 4.2. Analysis of pH and Dry Matter Content in FGM

The pH was measured with a pre-calibrated Milwaukee MW101 PRO pH meter (Milwaukee Instruments, Inc., Rocky Mount, NC, USA). Dry matter content was determined using the gravimetric method [54,55,56]. Analyses were performed as three replicates.

### 4.3. Analysis of HCB Content

The samples were first subjected to lyophilization using a LYOLAB 3000 freeze-dryer (Fisher Scientific, Loughborough, Leicestershire, UK) at a temperature of −60 ± 2 °C under reduced pressure. The lyophilized samples were extracted in a Soxhlet apparatus using a solvent mixture of hexane/acetone (*v*/*v*, 3/1, 150 mL) over an eight-hour period. The extracts were subsequently concentrated using a rotary vacuum evaporator (Büchi Rotavapor R-300 with Büchi B-300 Base heating bath; BÜCHI Labortechnik AG, Flawil, Switzerland) to a final volume of approximately 2 mL. The concentrated extracts were then quantitatively transferred into 10 mL vials and further reduced to approximately 0.5 mL under a stream of nitrogen.

**Figure 5:**
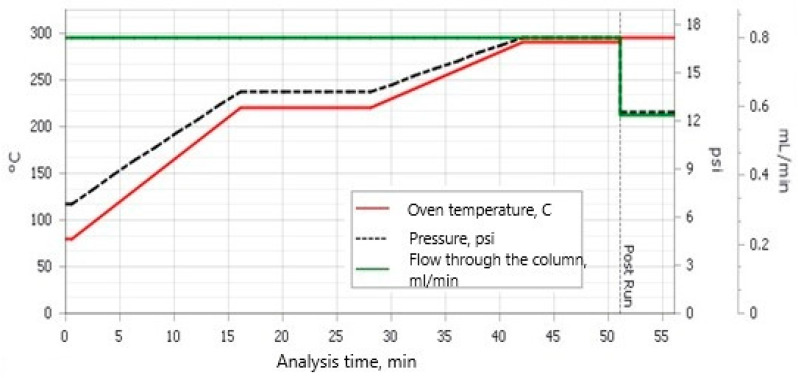
Chromatographic separation conditions [39].

The analytes were purified using 7% oleum (containing 7% SO_3_ in concentrated H_2_SO_4_). After phase separation, samples were further purified on glass LiChrolut columns (Merck, Darmstadt, Germany) packed with 1.5 g of silica gel. The samples were then concentrated under nitrogen and transferred to 1.5 mL chromatographic vials. The HCB residue was quantified by gas chromatography–mass spectrometry (GC/MS) using an Agilent 8890 GC system coupled with a 5977B Mass Selective Detector (Agilent, Santa Clara, CA, USA) and a DB-5 column (30 m × 250 µm × 0.25 µm). Helium 6.0 was used as the carrier gas, and a 2 µL sample injection volume was employed. The chromatographic separation conditions were as follows (Figure 5):

♦ 80 °C/0.5 min, ramp rate—9 °C/min to 220 °C (12 min),
♦ 220 °C/5 min, ramp rate—5 °C/min to 290 °C (9 min),
♦ column warming 295 °C/5 min,
♦ column hold at 295 °C/5 min.

**Figure 6:**
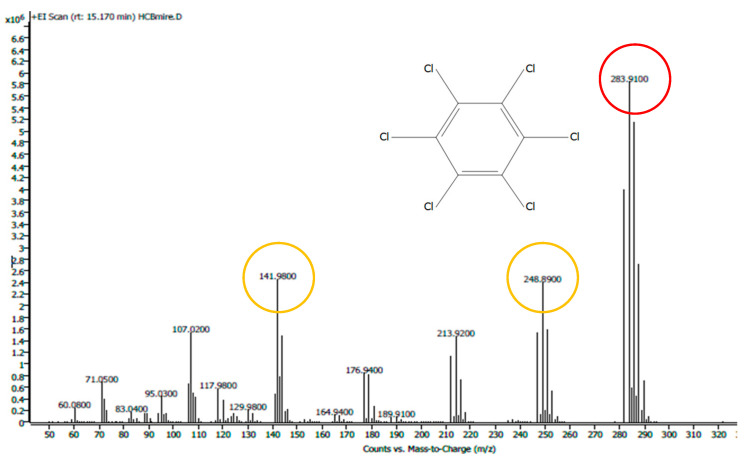
Mass spectrum of HCB [39]. Yellow—confirmation ions, red—the molecular ion.

Each sample was analyzed for 49.06 min as three replicates. Identification was performed based on the full mass spectrum (Figure 3). The HCB was quantified by analyzing both the test samples and calibration standard solutions using the mass spectrometer in Selected Ion Monitoring (SIM) mode (target ion [*m*/*z*] 283.91; confirmation ions: 248.89 and 141.98) (Figure 6). Figure 6 shows the full mass spectrum used to identify the compound hexachlorobenzene (HCB). Selected confirmation ions (yellow) and the molecular ion (red) are marked in color in circles.

### 4.4. Statistical Analysis

Statistical analysis was performed using STATISTICA 13.3 software (version 14.1.0 (32-/64-bit)/June 2023). The significance of differences was calculated using Duncan’s test, assuming a significance level of *p* < 0.05. Pearson’s correlation coefficient was also calculated.

## 5. Conclusions

The use of probiotic bacterial monocultures or mixtures of probiotic bacteria resulted in significant (*p* < 0.05) reductions in HCB content in fermented goat milk (FGM) during storage. All inoculation cultures achieved similar percentage reductions in HCB after 21 days of storage, ranging from 75% to 78%, with the *Lactiplantibacillus plantarum* monoculture showing marginally greater effectiveness. Additionally, strong positive correlations were observed between acidity and changes in HCB content among the FGM variants during refrigerated storage (r = 0.79–0.89).

Our findings from this pilot study confirm that the tested bacterial cultures could provide an alternative method for reducing HCB residues in fermented goat milk. Although none of the tested microbial cultures ensured complete biodegradation of HCB, they did contribute to a significant reduction in HCB content, potentially increasing the safety of fermented dairy products. Furthermore, our results underscore the need for further research to understand the emerging metabolites in food.

## Figures and Tables

**Table 2:** The pH and dry matter content of fermented goat milk (FGM) with and without added HCB during refrigerated storage (5 °C ± 1 °C).

Sample Variant		Storage Time (Days)			
		1	7	14	21
**pH ^1^**					
Without added HCB	LP	4.51 ± 0.02 ^aA^	4.31 ± 0.02 ^bA^	4.29 ± 0.01 ^cA^	4.29 ± 0.01 ^cA^
	LR	4.50 ± 0.01 ^aA^	4.30 ± 0.01 ^bA^	4.28 ± 0.01 ^cA^	4.28 ± 0.02 ^cA^
	Mix	4.48 ± 0.01 ^aA^	4.28 ± 0.01 ^bA^	4.28 ± 0.02 ^bA^	4.28 ± 0.01 ^bA^
With added HCB	LP_HCB_	4.53 ± 0.02 ^aA^	4.37 ± 0.02 ^bB^	4.30 ± 0.02 ^cA^	4.27 ± 0.02 ^dA^
	LR_HCB_	4.52 ± 0.03 ^aA^	4.32 ± 0.01 ^bA^	4.30 ± 0.01 ^cA^	4.26 ± 0.01 ^dA^
	Mix_HCB_	4.53 ± 0.02 ^aB^	4.34 ± 0.02 ^bB^	4.30 ± 0.02 ^cA^	4.26 ± 0.02 ^dA^
**Dry matter (%) ^1^**					
Without added HCB	LP	10.20 ± 0.02 ^aA^	10.20 ± 0.03 ^aA^	10.20 ± 0.04 ^aA^	10.20 ± 0.04 ^aA^
	LR	9.73 ± 0.03 ^aA^	9.73 ± 0.03 ^aA^	9.73 ± 0.03 ^aA^	9.73 ± 0.03 ^aA^
	Mix	10.13 ± 0.04 ^aA^	10.13 ± 0.04 ^aA^	10.13 ± 0.02 ^aA^	10.13 ± 0.04 ^aA^
With added HCB	LP_HCB_	10.28 ± 0.03 ^aB^	10.28 ± 0.02 ^aB^	10.28 ± 0.03 ^aB^	10.28 ± 0.02 ^aB^
	LR_HCB_	9.73 ± 0.02 ^aA^	9.73 ± 0.04 ^aA^	9.73 ± 0.04 ^aA^	9.73 ± 0.03 ^aA^
	Mix_HCB_	10.12 ± 0.02 ^aA^	10.12 ± 0.03 ^aA^	10.12 ± 0.02 ^aA^	10.12 ± 0.04 ^aA^

^1^ Arithmetic mean ± standard deviation; lowercase letters—significant differences (*p* < 0.05) during storage in individual variants of fermented beverages; capital letters—significant differences (*p* < 0.05) between samples inoculated with the same strain (in pair-wise comparison samples with and without HCB).

## Data Availability

Data are contained within the article.

## Supplementary Materials

The following supporting information can be downloaded at: https://www.mdpi.com/article/10.3390/molecules29235686/s1, Table S1: Changes in HCB content in fermented goat milk during refrigerated storage (5 °C ± 1 °C).
