# Supplementary material for: The Effect of Lactiplantibacillus plantarum and Lacticaseiba-cillus Rhamnosus Strains on the Reduction of Hexachlorobenzene Residues in Fermented Goat Milk During Refrigerated Storage
Source: Molecules. 2024 Nov 30;29(23):5686. doi: 10.3390/molecules29235686 (PMC11643662; doi:10.3390/molecules29235686)
Supplement: Supplementary file 1 [file molecules-29-05686-s001.zip › molecules-3339504-supplementary.pdf]

Table A1. Changes in HCB content in fermented goat milk during refrigerated storage (5°C ± 1°C).

Refrigerated storage period	Sample variant		HCB content in FGM without HCB	HCB content in FGM with HCB (93.5 ng/mL)	Average HCB reduction
			HCB concentration, ng/mL		
Day 1	LP[1]	x	0.06	87.56	6.35%
		SD	0.003	1.34	
		Me	0.06	87.31	
		RSD	0.05	0.015	
	LR[2]	x	0.086	89.31	4.48%
		SD	0.005	2.05	
		Me	0.087	89.54	
		RSD	0.058	0.029	
	Mix[3]	x	0.06	85.97	8.06%
		SD	0.002	2.45	
		Me	0.064	85.29	
		RSD	0.033	0.028	
Day 7	LP	x	0.094	80.19	14.23%
		SD	0.003	1.96	
		Me	0.095	81.04	
		RSD	0.032	0.024	
	LR	x	0.101	76.97	17.68%
		SD	0.005	2.07	
		Me	0.102	76.33	
		RSD	0.050	0.027	
	Mix	x	0.094	66.01	29.40%
		SD	0.002	1.88	
		Me	0.093	66.74	
		RSD	0.021	0.028	
Day 14	LP	x	0.087	26.83	71.30%
		SD	0.003	1.02	
		Me	0.086	26.97	
		RSD	0.034	0.038	
	LR	x	0.031	22.48	75.95%
		SD	0.002	1.34	
		Me	0.032	22.50	
		RSD	0.065	0.059	
	Mix	x	0.027	26.10	72.09%
		SD	0.003	1.17	
		Me	0.026	26.16	
		RSD	0.111	0.045	
Day 21	LP	x	0.087	20.36	78.23%
		SD	0.003	2.08	
		Me	0.088	20.48	
		RSD	0.034	0.102	
	LR	x	0.031	21.83	76.66%
		SD	0.002	1.45	
		Me	0.033	22.15	
		RSD	0.065	0.066	

Mix	x	0.027	22.52	75.92%
	SD	0.002	2.22	
	Me	0.026	22.43	
	RSD	0.074	0.099	

FGM—fermented goat milk; [1] LP—*Lactiplantibacillus plantarum*; [2] LR—*Lactocaseibacillus rhamnosus*; [3] Mix—*Lactiplantibacillus plantarum* and *Lactocaseibacillus rhamnosus.*
